# Local and systemic effects of fibrin and cyanoacrylate adhesives on lung lesions in rabbits

**DOI:** 10.6061/clinics/2017(10)06

**Published:** 2017-10

**Authors:** Marcus V.H. Carvalho, Evaldo Marchi, Andre J. Fruchi, Bruno V.B. Dias, Clovis L. Pinto, Geovane R. dos Santos, Milena M.P. Acencio

**Affiliations:** I Departamento de Cirurgia Toracica, Faculdade de Medicina de Jundiai, Jundiai, SP, BR; II Departamento de Patologia, Faculdade de Medicina de Jundiai, Jundiai, SP, BR; III Laboratorio de Pleura, Divisao Pulmonar, Instituto do Coracao (InCor), Hospital das Clinicas HCFMUSP, Faculdade de Medicina, Universidade de Sao Paulo, Sao Paulo, SP, BR

**Keywords:** Thoracic Surgery, Cyanoacrylate, Fibrin, Tissue Adhesives, Systemic Effects

## Abstract

**OBJECTIVES:**

Tissue adhesives can be used to prevent pulmonary air leaks, which frequently occur after lung interventions. The objective of this study is to evaluate local and systemic effects of fibrin and cyanoacrylate tissue adhesives on lung lesions in rabbits.

**METHODS:**

Eighteen rabbits were submitted to videothoracoscopy + lung incision alone (control) or videothoracoscopy + lung incision + local application of fibrin or cyanoacrylate adhesive. Blood samples were collected and assessed for leukocyte, neutrophil and lymphocyte counts and interleukin-8 levels preoperatively and at 48 hours and 28 days post-operatively. After 28 days, the animals were euthanized for gross examination of the lung surface, and lung fragments were excised for histopathological analysis.

**RESULTS:**

Fibrin and cyanoacrylate produced similar adhesion scores of the lung to the parietal pleura. Microscopic analysis revealed uniform low-cellular tissue infiltration in the fibrin group and an intense tissue reaction characterized by dense inflammatory infiltration of granulocytes, giant cells and necrosis in the cyanoacrylate group. No changes were detected in the leukocyte, neutrophil or lymphocyte count at any time-point, while the interleukin-8 levels were increased in the fibrin and cyanoacrylate groups after 48 hours compared with the pre-operative control levels (*p*<0.01).

**CONCLUSION:**

Both adhesive agents promoted normal tissue healing, with a more pronounced local inflammatory reaction observed for cyanoacrylate. Among the serum markers of inflammation, only the interleukin-8 levels changed post-operatively, increasing after 48 hours and decreasing after 28 days to levels similar to those of the control group in both the fibrin and cyanoacrylate groups.

## INTRODUCTION

Prolonged air leak (PAL) after lung resections is a frequent and challenging complication, increasing postoperative morbidity and the duration of hospital stays and resulting in high costs to the health system 1,2. When suturing and stapling fail to prevent air leaks, topical sealants may be considered 3-6.

Fibrin (FB) sealants have been used for more than a decade in lung resections, but their effectiveness is debated 3,4. In contrast, cyanoacrylate (CA) has been demonstrated to have a strong capacity for bonding tissues together 5,6; however, it is considered highly reactive to tissues and does not degrade over time 7.

To our knowledge, no literature addresses the systemic effects of tissue adhesives applied to lung lesions. This study aims to evaluate the local and systemic effects of the application of FB and CA tissue adhesives in an experimental model using videothoracoscopy (VATS) in rabbits with lung lesions.

## MATERIALS AND METHODS

This study was performed in accordance with human and animal care statements and Brazilian National Research Council guidelines for animal care, and it was approved by the Ethical Committee for Animal Research of Jundiai Medical School. Eighteen adult male New Zealand rabbits weighing 2.5 to 3.0 kg were randomized into three groups of six rabbits each. Each rabbit was anesthetized with 35 mg/kg of ketamine hydrochloride (Cristalia, Itapira, SP, Brazil) and 5 mg/kg of Xilazine (Bayer, Sao Paulo, SP, Brazil) that was infused through a catheter placed into the auricular vein. The animals were then positioned in the left lateral decubitus position, and their right chest areas were shaved and cleansed with iodopovidone (Rioquimica, S.J. Rio Preto, SP, Brazil). A mask adapted to each rabbits muzzle provided continuous oxygen during the entire experiment. Each rabbit underwent a videothoracoscopy procedure involving a lateral 2 cm skin incision in the right thorax, followed by muscle division and the intercostal insertion of a 5 mm trocar (Thoracoport, Medline, IL, USA) for accessing the right pleural cavity. Another 2 cm medial incision was made to introduce the second port for the lung incision procedure.

The treatment groups were as follows: a control group (VATS + lung incision without treatment), an FB group (VATS + lung incision + FB adhesive application - Evicel^®^; Omrix Biopharmaceuticals, Ethicon, Israel) and a CA group (VATS + lung incision + CA adhesive application - Omnex^®;^ Omrix Biopharmaceuticals, Ethicon, Israel). All lung incisions were 1 cm in length and performed in the middle lobe. The amount of sealant applied to each lung incision was 1 mL.

Before the trocars were withdrawn, the residual air in the pleural space was completely aspirated, and the skin was sutured with 4-0 monofilament nylon. During the experiment, the animals were carefully monitored using pulse oximetry and electrocardiography. The same surgeon and surgical team performed all surgical interventions.

### Euthanasia and Macroscopic Analysis

On the 28^th^ postoperative day, the animals were anesthetized and euthanized with a lethal dose of pentobarbital. Each thorax was removed *en bloc,* and 60 mL of 10% formalin was infused through the trachea to maintain lung expansion. The thorax was maintained in the formalin solution for 48 hours and subsequently opened by a longitudinal sternal incision to access both pleural cavities for macroscopic examination. Adhesions between the visceral and parietal pleura were scored 8 based on a 1-4 grade scale, as follows: 1 - no adhesions; 2 - fewer than three localized adhesions; 3 - more than three localized adhesions, and 4 - complete obliteration of the pleural cavity.

### Leukocyte, Neutrophil and Cytokine Measurements

Blood samples were collected preoperatively and 48 hours and 28 days after surgery to evaluate the systemic effects of the interventions. Total blood leukocytes and neutrophils were measured by an automated hematological analyzer that included a specific module for rabbit blood analysis (ADVIA, Bayer, Germany). Blood samples were centrifuged at 1,000 rpm for 10 minutes at 4° C. The supernatant was removed and stored at -80° C for later quantification of the cytokine interleukin-8 (IL-8) (Pharmingen, San Diego, CA); quantification was performed by ELISA using a 450 nm Power Wave filter (Bio-Tek, Winooski, VT, USA). The minimum detection level for IL-8 was 15.6 pg/mL.

### Histopathological Analysis

For microscopic analysis, lung tissue from the operated area was sectioned, fixed in 10% buffered formalin and embedded in paraffin. The 3-mm-thick sections were processed and stained with hematoxylin-eosin (HE) for microscopic analysis. To identify collagen fibers in the visceral pleural samples, the slides were stained with 2% Sirius red solution dissolved in aqueous saturated picric acid. By this method, the thin fibers (immature collagen, typed III) stain green, while the thick fibers (mature collagen, type I) stain orange-red. The same pathologist analyzed the samples in a blinded fashion.

## RESULTS

All procedures were well tolerated, and all animals were stable with respect to hemodynamics and oxygen saturation during the interventions. There were no deaths at any point of the study in the groups, including the control group, which received a lung lesion without any treatment.

### Macroscopic Findings

No difference in the medium scores of pleural adhesions was observed in the control, FB or CA group. However, the CA group produced slightly more pleural adhesions than did the control group (*p=*0.051).

**Figure 1:**
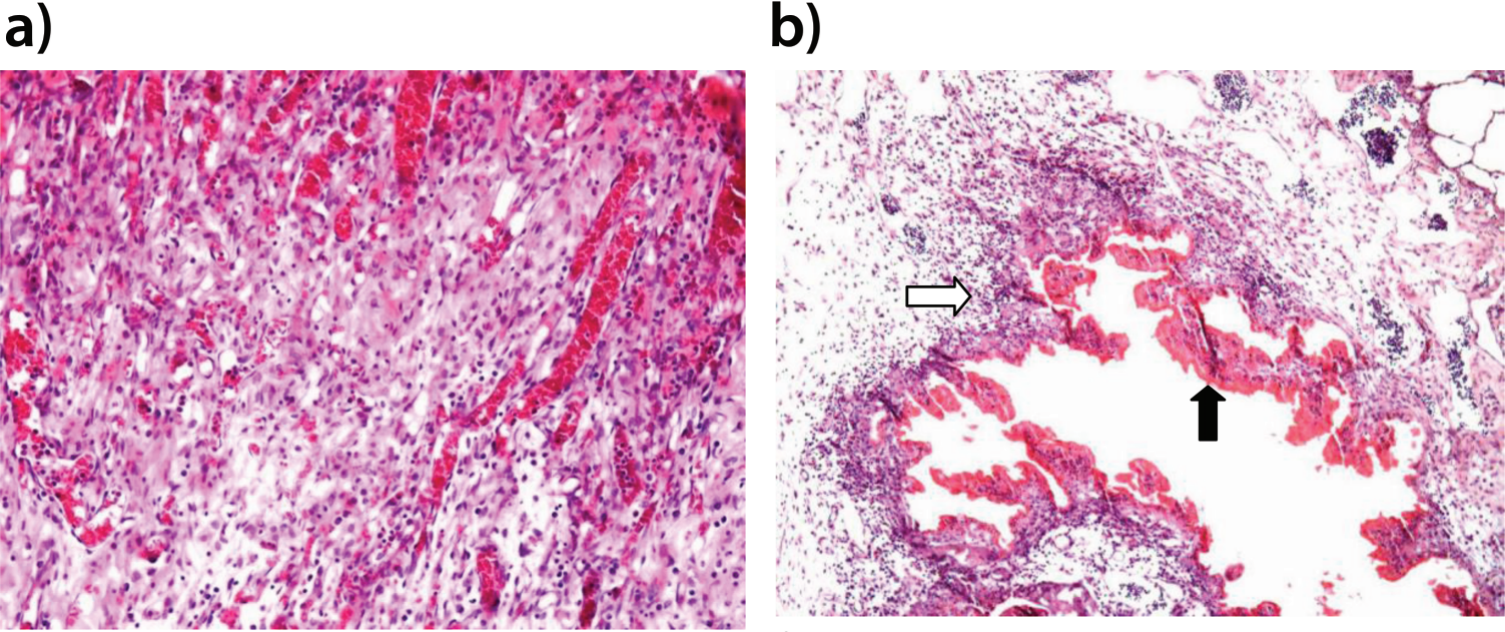
Photomicroscopy image of rabbit lung parenchyma stained by HE (X100 magnification). The image in **a** (FB group) shows intense neoangiogenesis and inflammatory cell infiltration, which are findings compatible with normal wound healing. The image in **b** (CA group) shows a palisade of histiocytic cells and lymphocytes (white arrow) and a central area of necrosis (black arrow).

The macroscopic examination at 28 days post-operatively revealed that no residual FB adhesive remained on the lung surface and that the operated area was whitish, with no texture modification. In contrast, at 28 days postoperatively in the CA group, a hard consistency of the lung parenchyma was observed, and residual adhesive was present over an area of approximately 0.5 cm around the application site (Figure 1).

### Histological Analysis

**Figure 2:**
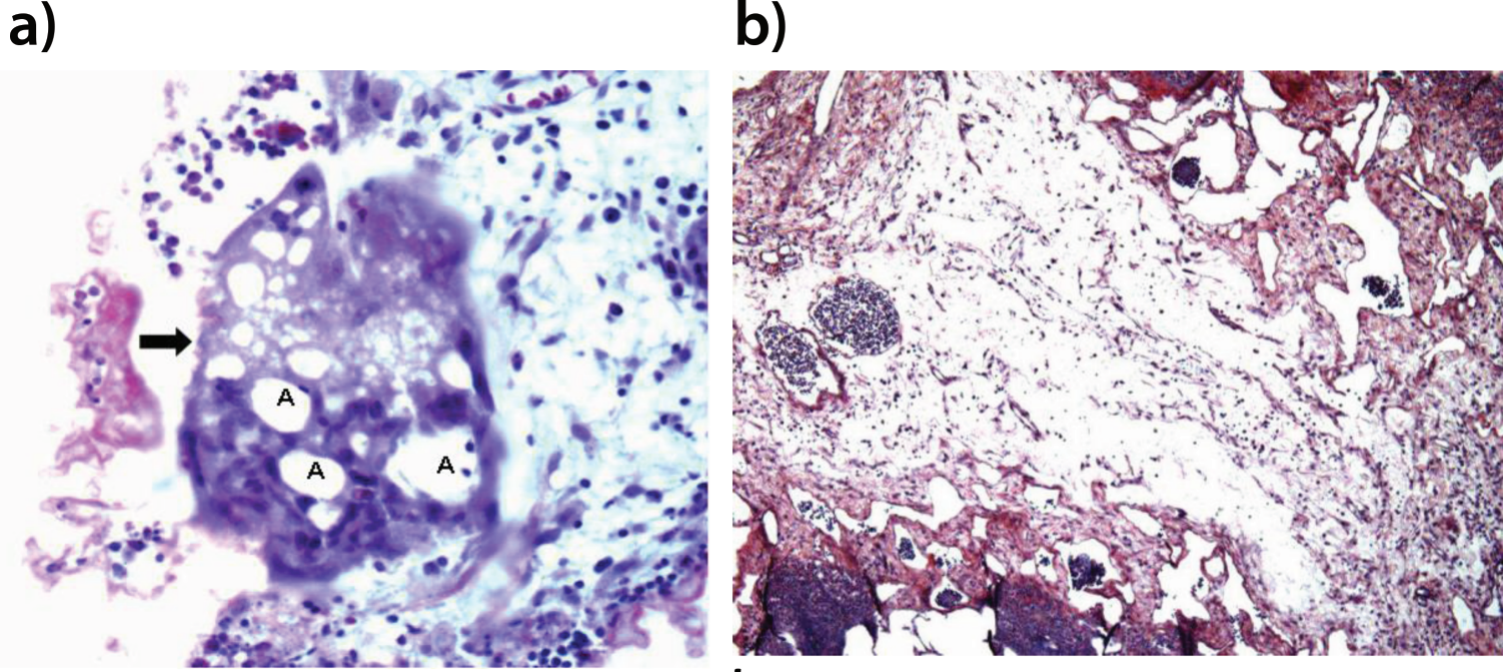
Photomicroscopy image of rabbit lung parenchyma from the CA group. The image in **a** shows a multinucleated giant cell (arrow) with cytoplasmic vacuoles containing CA residues with HE staining (X400 magnification) (A). The image in **b** shows an absence of collagen deposits and accentuated stromal edema with picrosirius red staining (X100 magnification).

For the histological analysis, the FB group presented with locally rich neo-angiogenesis and a mix of inflammatory infiltration and edema, similar to that observed in the normal phases of scar formation (Figure 1a). In contrast, in the CA group, a dense lymph-mononuclear inflammatory infiltration with lymphoid aggregates was observed, permeated by cavitation areas. Covering the cavitation areas, a histiocytic ‘in palisade’ infiltration was observed, as were some multinucleated giant cells with cytoplasmic vacuolization and residues of phagocytosed CA adhesive (Figure 1b and 2a). The interior of the cavities presented with an amorphous content and necrosis. In addition, the perilesional stroma showed edema with minimum deposition of collagen fibers (Figure 2b).

### Systemic Effects

There was no significant variation in the total leukocyte, neutrophil or lymphocyte count in the pre-operative phases or 48 hours or 28 days post-operatively in any group (Table 1). Serum IL-8 levels were significantly elevated after 48 hours in both the FB and CA groups compared with those in the control group (*p*<0.01). However, at 28 days postoperatively, the IL-8 levels in both groups had decreased to levels comparable to those of the control group.

## DISCUSSION

The incidence of intra-operative air leak may be as high as 60 to 70% in patients after lung resection 9-11, and it can be prolonged in approximately 8 to 15% of patients 12. The incidence of PAL depends on several factors, such as the presence of damaged or fragile lung parenchyma 13, the use of steroids 12-14, whether upper lobectomy is performed 15, and the presence of pleural adhesions 16. Thus, PAL remains a frequent and challenging complication. To help prevent postoperative lung air leakage, tissue sealants/adhesives can be used.

FB is the most common adhesive agent used in surgical practice. As a biological substance, FB allows adequate tissue healing, but concerns about its use have been raised, including its unclear efficiency 17,18 and the possibility of allergic reactions to its proteins 19. In contrast, CA is a synthetic product with known efficiency, although its use also raises concerns, specifically regarding biocompatibility and local toxicity 20.

The FB tissue adhesive consists of a combination of human plasma fibrinogen, thrombin and an anti-fibrinolytic product. In the presence of thrombin, fibrinogen is converted into a fibrin clot. FB glue has been the most widely used tissue adhesive in thoracic surgery over the past two decades. However, interest in the product has decreased since randomized trials conducted by Fleisher 17 and Wong 21. In these trials, the use of FB in comparison with the control revealed no differences in outcome, number of postoperative air leaks, chest drain duration or length of hospital stay. In a recent systematic review addressing the application of surgical sealants, primarily FB adhesives, after pulmonary resection, the authors concluded that there are decreases in postoperative air leaks and time to chest drain removal and no reduction in the length of postoperative hospital stay 22. One of the most important benefits of fibrin sealants is their low inflammation and rapid degradation compared with those of other sealants 23. Although some studies have shown that FB sealants allow a superior aerostasis in patients undergoing pulmonary resection 24, other studies have reported a lower resistance bonding property of FB sealants compared with that of CA glue 25.

CA adhesive has shown effectiveness in preventing air leaks, but its use is associated with intense local reactions and no signs of degradation over time 26,27. The current study found an intense local reaction to CA applied to lung lesions in rabbits; however, no systemic repercussion, as measured by the response of leukocytes and neutrophils, was observed. Nevertheless, both FB and CA produced an acute elevation of serum IL-8 levels 48 hours after application. To our knowledge, no previous studies have addressed the systemic effects of CA and FB sealants/adhesives applied to lung lesions.

Concerning the local reactions to adhesives, the findings of the present study are similar to those of previous reports 23,28. The reactions mainly include a pronounced local inflammatory response to CA, with the infiltration of granulocytes, lymphocytes and foreign body giant cells, and minor signs of degradation over time. However, when FB sealant was used, no residual adhesive was found and microscopy analysis revealed adequate tissue healing.

CA derivatives have low viscosity, dry rapidly, gain adhesion rapidly and, most importantly, have strong adhesive capability. Among several available commercial formulations, Omnex® represents a long-chain derivative of octyl-cyanoacrylate that polymerizes slowly and gradually releases heat to the injured tissue. In theory, newer formulations of CA are less toxic; however, they are not completely free of the adverse inflammatory properties of previous products. The findings of our study show that CA shows persistent adherence to lung tissue, and after drying, it polymerizes and becomes a very stable and inert compound. A rodent model of CA injection showed that this polymer persisted inside the abdominal cavity up to 12 months after local application 27.

Although CA does not appear to degrade over time, local toxicity does not appear to be a concern. In a study using electron microscopy, CA showed no inhibition of tissue regeneration and no histotoxicity 29. Another study with nanoparticles composed of CA revealed that the substance was stable and showed no toxicity 30. In addition, amnion cell cultures from fetal membranes showed no toxicity after exposure to CA 31. In another *in vitro* study using mouse fibroblasts, it was found that when a large number of cells was used, cell proliferation overcame the cytotoxic effect of CA 32. Another study with cardiomyoblasts using an extract dilution assessment showed that CA had no toxic effect on the cells 33.

Regarding the possible systemic inflammatory reaction related to the application of FB or CA to lung lesions, the present study did not find an increase in blood leukocyte, neutrophil or lymphocyte count following application. Montanaro L et al. 34 found no significant variation in the numbers of total or differential leukocyte counts when CA glue was used. In our study, although the blood cellular profile did not vary among groups, the serum levels of IL-8 increased 48 hours after the application of FB or CA in lung lesions. These results show that the local inflammatory reaction may produce systemic repercussions when more sensitive markers are used than when cellular counts alone are used. The IL-8 levels increased in a similar manner for the two adhesive agents, and the levels of this cytokine decreased at day 28 to levels comparable to those of the control group.

In conclusion, either FB or CA applied to the lung lesions of rabbits promoted effective local healing. CA promoted a pronounced local inflammatory response with cell infiltration and the appearance of foreign body giant cells, and FB promoted normal tissue healing.

The only systemic effect observed for both agents was an elevation of serum IL-8 levels at 48 hours; these levels had decreased at 28 days after the procedure. These findings may be relevant to surgeons who need to use adhesives as adjuvants to seal complex injuries. In addition, the current study may decrease the concern regarding the potential undesirable adverse effects of CA that have been reported by previous studies.

## AUTHOR CONTRIBUTIONS

Carvalho MV and Marchi E conceived and designed the study, they were responsible for the animal procedures, the acquisition and analysis of the data, and the drafting and revision of the manuscript. Fruchi AJ collaborated in all phases of the study. Dias BV, Pinto CL, dos Santos GR and Acencio MM analyzed and interpreted the samples and helped in writing the manuscript.

## Figures and Tables

**Table 1:** Values (median and quartiles [25-75%]) of total leukocyte, neutrophil, and lymphocyte counts (cells/mm^3^) and IL-8 levels (pg/mL) at 48 hours and 28 days in the control, fibrin and cyanoacrylate groups.

		Control	Fibrin	Cyanoacrylate	*p* value
**Leukocytes**	**48 h**	8,800 (7,300 – 10,500)	9,100 (7,800 – 9,500)	6,800 (6,300 – 8,200)	0.138
	**28 d**	8,100 (6,100 – 9,400)	7,400 (7,000 – 8,800)	6,850 (6,700-7,100)	0.214
**Neutrophils**	**48 h**	4,168 (1,339 – 6,997)	3,491 (2,380 – 4,602)	3,664 (1,599 – 4,775)	0.745
	**28 d**	3,413 (2,040 – 4,050)	3,300 (2,170-4,361)	3,146 (1,800 – 3,645)	0.846
**Lymphocytes**	**48 h**	3,868 (3,504 – 4,800)	4,940 (3,666 – 5,096)	5.126 (4.284 – 6.120)	0.085
	**28 d**	4,087 (3,888-4,753)	3,784 (3,774-4,183)	3,070 (3,932-4,761)	0.450
**IL-8**	**48 h**	38.6 (38.5-49.6)	75.3 (67.3-86.0)	72.2 (64.5-83.5)	**<0.010**
	**28 d**	69.1 (32.3-72.9)	78.4 (49.8-80.3)	67.6 (28.3-79.1)	0.297

There was a significant difference in the IL-8 level between the control and treatment groups at 48 hours but not at 28 days (*p*<0.010). No difference was found in the leukocyte, neutrophil or lymphocyte count.
